# The incidence and pattern of copollinator diversification in dioecious and monoecious figs

**DOI:** 10.1111/evo.12584

**Published:** 2015-01-19

**Authors:** Li-Yuan Yang, Carlos A Machado, Xiao-Dong Dang, Yan-Qiong Peng, Da-Rong Yang, Da-Yong Zhang, Wan-Jin Liao

**Affiliations:** 1 State Key Laboratory of Earth Surface Processes and Resource Ecology and MOE Key Laboratory for Biodiversity Science and Ecological Engineering, College of Life Sciences, Beijing Normal UniversityBeijing, 100875, China; 2 Department of Biology, University of Maryland1210 Biology-Psychology Building, College Park, Maryland, 20742; 3 Key Laboratory of Tropical Forest Ecology, Xishuangbanna Tropical Botanical Garden, Chinese Academy of SciencesKunming, 650223, China

**Keywords:** Breeding system, cospeciation, *Ficus*, fig-pollinating wasp, host specificity, host switching

## Abstract

Differences in breeding system are associated with correlated ecological and morphological changes in plants. In *Ficus*, dioecy and monoecy are strongly associated with different suites of traits (tree height, population density, fruiting frequency, pollinator dispersal ecology). Although approximately 30% of fig species are pollinated by multiple species of fig-pollinating wasps, it has been suggested that copollinators are rare in dioecious figs. Here, we test whether there is a connection between the fig breeding system and copollinator incidence and diversification by conducting a meta-analysis of molecular data from pollinators of 119 fig species that includes new data from 15 Asian fig species. We find that the incidence of copollinators is not significantly different between monoecious and dioecious *Ficus*. Surprisingly, while all copollinators in dioecious figs are sister taxa, only 32.1% in monoecious figs are sister taxa. We present hypotheses to explain those patterns and discuss their consequences on the evolution of this mutualism.

Interspecific mutualisms between flowering plants and their insect pollinators represent one of the most influential types of biological interaction (Herre et al. 1999). The evolution of these mutualisms has played a major role in the radiation of flowering plants, and has had a major influence in the evolution and maintenance of biodiversity. One of the most tightly integrated pollination mutualisms known occurs in the obligate relationship between figs (*Ficus* spp.) and their pollinating wasps (Agaonidae, Chalcidoidea) (Janzen 1979; Herre et al. 2008). Each species of fig depends on one or more highly specialized fig wasp species that pollinate its flowers. In turn, the wasps depend on the fig for the production of offspring and for completing their life cycles. About half of the approximately 750 described species of *Ficus* are monoecious, the ancestral breeding system in figs, whereas the other half are dioecious (Berg 1989), a breeding system that has evolved from monoecious ancestors at least twice in this genus with one possible reversal (Jousselin et al. 2003; Herre et al. 2008). The observed variation in breeding system in *Ficus* poses interesting questions about what evolutionary pressures may have influenced the inferred transitions from monoecy to dioecy or back, and whether those changes may have influenced patterns of evolutionary diversification in both mutualists.

Differences in breeding system are associated with correlated ecological and morphological changes in plants, pollination mechanisms, and growth mode being the traits showing the most consistent associations (Renner and Ricklefs 1995). In *Ficus*, dioecy and monoecy are strongly associated with different traits. Monoecious *Ficus* are tall trees that reach the canopy, with very low population densities (<1 individual per hectare). Further, monoecious figs show low levels of genetic differentiation (Kobmoo et al. 2010; Nazareno et al. 2013), consistent with the observation and inference of long-distance pollination (Nason et al. 1998; Compton et al. 2000; Harrison 2003; Zavodna et al. 2005; Harrison and Rasplus 2006; Ahmed et al. 2009). On the other hand, dioecious *Ficus* are usually small shrub-like trees that rarely reach the canopy, with high local population densities, and that produce fruit more frequently than monoecious trees (Harrison and Yamamura 2003). In dioecious species, some individuals are functionally male, only producing pollen-carrying wasp progeny, whereas others are functionally female and only produce seed-bearing fruit (female pollinators entering a female fig cannot lay eggs; Janzen 1979). Pollinators of dioecious *Ficus* show limited ranges of pollen dispersal (Harrison 2003; Harrison and Rasplus 2006) that are thought to generate the significant patterns of spatial genetic structure normally observed in these fig species (Wang et al. 2009; Chen et al. 2011; Dev et al. 2011; Nazareno et al. 2013; but see Yu et al. 2010 for an exception).

Interestingly, differences in breeding system in *Ficus* also seem to correlate with patterns of pollinator association. Although this mutualism has been usually described as having highly reciprocal one-to-one pollinator–host species specificity, this notion has been challenged by the observation that a significant fraction of fig species examined have more than one species of pollinator (copollinators), and that even some fig species share pollinators (reviewed in Herre et al. 2008; Cook and Segar 2010). Intriguingly, it has been suggested that copollinators are rare in dioecious figs (Cook and Segar 2010; Moe et al. 2011), raising the possibility that the occurrence of copollinators may be influenced by the fig breeding system. Potential differences in the prevalence of copollinators could reflect biological differences between mutualists of the two breeding systems, although sampling biases may also be involved because most molecular surveys of pollinators have been conducted in areas with a prevalence of monoecious figs (America, Africa; Cook and Segar 2010). Nevertheless, this apparent pattern raises interesting questions about the effect of breeding system on fig and fig wasp diversification that no studies have yet addressed. First, it is crucial to include data from new molecular surveys of pollinators that include a larger sample of dioecious figs to properly test if the suggested pattern of differences in copollinator prevalence between the two breeding systems is real and not the result of sampling biases. More important, however, is to address whether patterns of copollinator diversification (i.e., speciation within the same fig host [duplication] vs. pollinator host switches) are different across fig breeding systems. This is a key question because it can provide insights into the effect of breeding system on expected coevolutionary patterns in the mutualism, on patterns of gene flow between fig species, and may also help understand what evolutionary forces could have influenced transitions between breeding systems in *Ficus*.

One characteristic of fig pollinating wasps that is correlated with the fig breeding system could influence copollinator prevalence and diversification: pollinator dispersal ecology (Compton et al. 2000; Harrison 2003; Harrison and Rasplus 2006). Pollinators of monoecious figs show long-distance dispersal (Nason et al. 1998; Harrison 2003; Zavodna et al. 2005; Harrison and Rasplus 2006; Ahmed et al. 2009), whereas pollinators of dioecious *Ficus* disperse over small distances (Harrison 2003; Harrison and Rasplus 2006; Wang et al. 2009; Chen et al. 2011; Dev et al. 2011; Nazareno et al. 2013). Those differences in pollinator dispersal may be important for explaining expected patterns of copollinator incidence. For instance, the fact that the pollinators of monoecious *Ficus* travel longer distances to find receptive host trees makes them more likely to encounter hosts from more fig species than pollinators of dioecious figs. For that reason they could be more likely to make host recognition mistakes, and one thus could predict that host switches would be more common in pollinators of monoecious figs. However, it could also be argued that even though dioecious fig pollinators disperse across shorter distances, they still come across a large number of sympatric fig species given the high population densities of dioecious *Ficus*, and could therefore still be prone to making host recognition mistakes. However, if there are significant differences between the two breeding systems in the strength of species-recognition mechanisms and/or in the fitness costs paid by pollinators for making host identification mistakes, then the incidence of host identification mistakes and host switches will strongly depend on the fig breeding system. For instance, if fitness costs for host identification mistakes were higher in dioecious figs (Moe and Weiblen 2012), then pollinator host switches should be more common in monoecious figs (Machado et al. 2005). None of those predictions have been tested.

Here, we present results documenting the occurrence of copollinators and their patterns of phylogenetic association in a group of 10 dioecious and five monoecious sympatric fig species from southwest China, and combine those results with all published molecular studies of the pollinators of 104 additional species of *Ficus* (119 species in total) to conduct a meta-analysis. We address two main questions: (1) Do monoecious and dioecious figs differ in their observed incidence of co-pollinators? (2) Are patterns of copollinator diversification different between fig breeding systems? That is, are copollinators more likely to be sister or nonsister species depending on the fig breeding system? These novel results provide important insights for understanding the influence of breeding system on fig and fig wasp diversification.

## Materials and Methods

### Collections and Sequence Data

Pollinators from 15 locally abundant fig species (10 dioecious and five monoecious) were collected from a 1.5 km^2^ area of tropical rainforest in Xishuangbanna, China (21°41′N, 101°25′E; Table S1). Foundress wasps were collected after they entered receptive enclosed fig inflorescences (syconia) and stored in 95% ethanol at −20°C. All pollinators were collected from 10 to 40 receptive syconia from at least three different host trees of each species. In total, 16–41 fig-pollinating wasp individuals were sampled from each host fig species for a total of 369 fig wasp individuals.

Sequence data were collected from the 3′′ end of the *COI* mitochondrial gene and the D2 domain of the 28S rRNA genes, using standard methods. These gene regions have been frequently used in phylogenetic studies of closely related species of fig wasps (e.g., Molbo et al. 2003; Cruaud et al. 2012). The entire genomic DNA was extracted from the ethanol-preserved individuals, using the E.N.Z.A.™ Insect DNA Kit (OMEGA, Norcross, GA) according to manufacturer's instructions. The 3′ end of the *COI* gene and the D2 domain of the 28S rRNA gene were polymerase chain reaction (PCR) amplified, using the Jerry and Pat primers (Simon et al. 1994) and the D1F and D3R primers (Lopez-Vaamonde et al. 2001), respectively, using standard conditions. The PCR products were gel extracted using the Gel Extraction Mini Kit (Watson, China) and directly sequenced using the ABI PRISM BigDye Terminator v 3.1 Ready Reaction Cycle Sequencing Kit (Applied Biosystems, Foster City, CA). A total of 357 *COI* sequences were collected, representing 168 unique haplotypes; 170 28S rRNA sequences were collected, representing 19 unique haplotypes. All unique haplotype sequences were deposited in GenBank (accession numbers HM802558-HM802653, HM802669-HM802758, HQ456879).

### Phylogenetic Analyses

The sequences of the *COI* gene were aligned using multiple alignment using fast fourier transform (MAFFT; Katoh and Standley 2013). The *COI* sequences were also translated into amino acids using MEGA 4 (Tamura et al. 2007) to detect frame-shift mutations and premature stop codons (neither were found). LocARNA (Will et al. 2012) was used for 28S rRNA gene sequence alignment based on secondary structure. We selected separate models of molecular evolution for the different sequence regions (*COI*, rRNA loops, and rRNA stems), using the Akaike information criterion implemented in Modeltest 3.7 (Nylander et al. 2004). The best models for each partition were GTR + I + G (*COI*), TVM + G (28S rRNA loops), and GTR + I + G (28S rRNA stems). Phylogenetic analyses were performed on the separate and combined datasets using both Bayesian and maximum likelihood methods. The Bayesian analyses were implemented in MrBayes 3.1.1 (Huelsenbeck and Ronquist 2001; Ronquist and Huelsenbeck 2003), using the Markov Chain Monte Carlo method with the best-fitting molecular evolution model and parameters of each gene. Two independent runs were implemented with 10 million generations. Each run included a cold chain and three heated chains with the heating temperature of 0.2. The current tree with branch lengths was saved every 100 generations. The initial 25% of the sampled trees were discarded as burn-in, and the trees collected after the burn-in point were used to construct the 50% majority-rule consensus tree. Maximum likelihood analyses were conducted in RAxML7.2.6 (Stamatakis 2006), using GTRGAMMA models to find the best tree, and 1000 bootstrap replicates were conducted. We used GenBank sequences from four Sycophaginae species (*Idarnes* sp.: JN001574, JN001505; *Apocryptophagus* sp.: JN001556, JN001502; *Odontofroggatia* sp.: HM770633, HM770695; *Anidarnes gracilis*: JQ925909, JQ925927) as outgroups in the phylogenetic analysis based on recent analyses showing that Agaoninae and Sycophaginae are sister groups (Heraty et al. 2013).

To determine whether the copollinators from our samples were potential sister taxa, we downloaded all published *COI* gene sequences of genera *Ceratosolen* and *Eupristina*. We combined those GenBank sequences with our data and reconstructed Bayesian phylogenetic trees for each genus using the same methods described above. The datasets consisted of 176 haplotypes from *Ceratosolen* (357bps) and 55 haplotypes from *Eupristina* (716 bps).

### Pairwise Distance Comparisons

For fig wasps with more than one subclade on the *COI* phylogenetic tree (*Ceratosolen emarginatus*, *C. gravelyi*, *Eupristina Koningsbergeri*, and *Blastophaga* sp. 3), we estimated *COI* sequence divergence between all pairs of individuals within each subclade and between the two subclades using the K2P distance model in MEGA (Tamura et al. 2007). Pairwise sequence divergences were not calculated for 28S rRNA because no sequence variation was observed within subclades. The use of this histogram method to delimit species using genetic data is well established in the literature (Sites and Marshall 2004).

### Copollinator Data Acquisition and Meta-Analysis

We gathered results from other published molecular studies to test differences in the occurrence of copollinators between monoecious and dioecious figs and to test whether patterns of copollinator diversification differ between fig breeding systems. We conducted 22 searches in the Web of Science database, using combinations of 11 keywords (Table S2). The searches resulted in a total of 133 articles, and the reference lists of those articles were also examined to identify other potentially relevant articles. In all, only 19 of the 133 articles presented molecular data from fig wasps that were relevant to the meta-analysis. Combined with our data, the meta-analysis encompassed results from 119 fig species (30 dioecious, 89 monoecious), four genera of pollinators from dioecious figs and 11 genera of pollinators from monoecious figs (Table S3). Data were classified according to the host fig breeding system, the number of copollinators (one or multiple), the phylogenetic relationships of copollinators (sister taxa or nonsister taxa), and geographic location (Table S4). Phylogenetic relationships of copollinators from a fig species were categorized as sister taxa if all copollinators formed a monophyletic clade, and as nonsister taxa if at least one species of copollinator was not closely related to the others. This choice ensured that the tests were conservative as only one datapoint per fig species was used. In addition, we also estimated the minimum number of duplication or host-switch events that can explain the phylogenetic relationships of the copollinators: each speciation within a host was counted as one duplication event, and each distantly related species of copollinator was counted as one host-switch event. Chi-square and Fisher exact tests were used to test the differences.

## Results

### Molecular Phylogeny and Sequence Divergence

**Figure 1:**
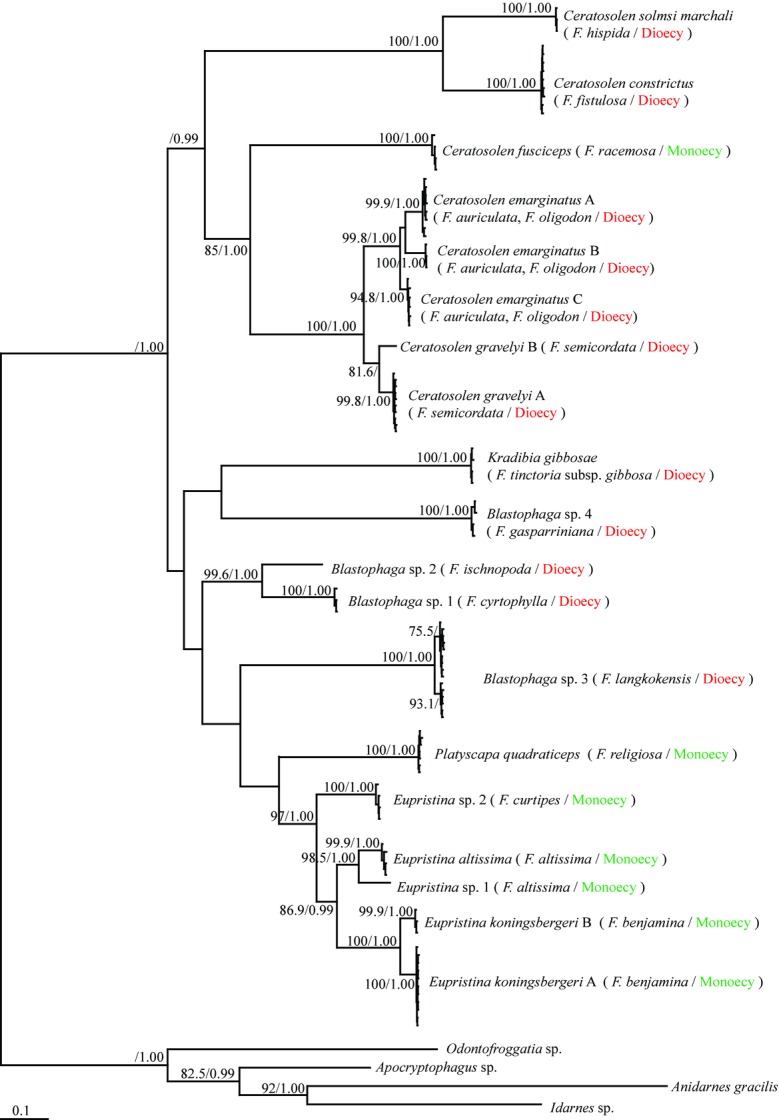
The combined *COI* and 28S rRNA Bayesian tree of fig pollinating wasps collected from 15 host figs. Maximum likelihood (ML) bootstrap percentages (>70%, 1000 replications) and Bayesian posterior probabilities (>0.95, 10^7^ generations) are indicated on the nodes. Fig host names and their breeding systems are indicated in parentheses.

**Figure 2:**
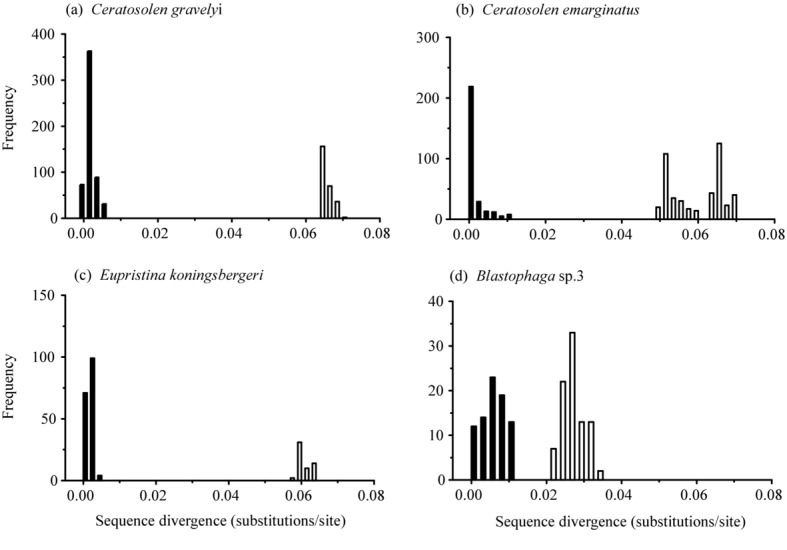
Pairwise *COI* sequence divergence histogram between individuals within each subclade (black bars) and between sister subclades (open bars) in *Ceratosolen gravelyi* (a), *C. emarginatus* (b), *Eupristina koningsbergeri* (c), and *Blastophaga* sp. 3 (d).

Phylogenetic analyses of the combined *COI* and 28S rRNA sequences from the newly surveyed 15 species show that all pollinator clades are strongly supported (Fig.1). Five putative cases of multiple local copollinators were observed, three in dioecious fig hosts and two in monoecious fig hosts (Fig.1). Deep *COI* gene sequence divergence between subclades (>4%) was observed in the pollinators of *Ficus semicordata* (*Ceratosolen gravelyi*, two clades), *F. benjamina* (*Eupristina koningsbergeri*, two clades), *F. altissima* (*Eupristina* sp. 1 and *E. altissima*), and *F. auriculata* and *F. oligodon*, which share the same pollinator (*C. emarginatus*, three clades; Fig.2). *COI* sequences were also obtained from multiple pollinating wasps that had completed their development in syconia of *F. auriculata* and *F. oligodon* (*C. emarginatus*) and in *F. semicordata* (*C. gravelyi*), showing the same subclades observed from the main sample of foundresses (Fig. S5). The 28S rRNA gene was less variable than the *COI* gene and was highly homogenous within species, but divergent between species. Intraspecies divergence in the 28S rRNA gene was only detected in three species: *C. gravelyi*, *C. emarginatus*, and *E. koningsbergeri*. Each subclade suggested by the *COI* phylogeny had one unique 28S rRNA haplotype (Figs. S1 and S2). Moreover, all copollinators from a fig species were sister species, as shown by additional phylogenetic analyses that included *COI* sequences from other species of the same genera downloaded from GenBank (Figs. S3 and S4).

The copollinators from *F. altissima* correspond to morphologically distinct species that were known in advance of our molecular survey (Peng et al. 2008). The average pairwise *COI* sequence divergence within each copollinator subclade of *C. emarginatus*, *C. gravelyi*, *E. koningsbergeri*, and *Blastophaga* sp. 3 was much lower than between subclades. In all four cases, pairwise divergence within a subclade was lower than 1.4%, whereas the average divergence between subclades was 5.2–7.4% in the case of *C. emarginatus*, *C. gravelyi*, and *E. koningsbergeri*, but only 2.4–3.6% in *Blastophaga* sp. 3 (Fig.2). In previous studies, the range of observed sequence divergence in the *COI* gene between cryptic fig wasp species pairs has been 4.2–6.6% in Panama (Molbo et al. 2003), 3.8–7.2% in Papua New Guinea (Moe et al. 2011), and 4.2–5.3% among three cryptic species of *Eupristina verticillata* in China (Sun et al. 2011). In all those cases, the divergence was larger than the divergence observed in *Blastophaga* sp. 3. Furthermore, *Blastophaga* sp. 3 showed no intraspecific divergence in the 28S rRNA gene (Fig. S2) and for that reason we do not include this species in our count of multiple copollinators (Table S3).

*Ficus auriculata* and *F. oligodon* share the same group of three copollinators: the three different sister clades of *C. emarginatus* (Fig.1 and Fig. S3). These two fig species are morphologically very similar, although they have been described as different species in Flora of China (Zhou and Gilbert 2003). However, recent ecological data (Y.-Q. Peng and D.-R. Yang, unpubl. ms.) suggest that they are the same species, a conclusion consistent with our observation of them sharing three identical pollinator species. Furthermore, recent molecular data for morphotypes of this species complex also confirm the presence of three closely related *Ceratosolen* pollinators (Wei et al. 2014).

### Meta-Analysis of Copollinators

We combined our new data with that from 19 published studies investigating pollinator species diversity using molecular methods to analyze copollinator presence and patterns of copollinator phylogenetic relationships in 89 monoecious fig species and 30 dioecious fig species (Table1 and Table S3). Overall, 34.5% of fig species (41/119) in the combined dataset have more than one pollinator species. Contrary to previous suggestions (Cook and Segar 2010; Moe et al. 2011), the proportion of fig species showing multiple pollinator species is not significantly different between monoecious (31.5%) and dioecious figs (43.3%; (χ^2^ = 1.40, *P* = 0.2366; Fisher's *P* = 0.2703). Rarefaction analysis was performed to evaluate the possible effects of unbalanced species sampling between breeding systems. We constructed 1000 pseudosamples with even sample sizes of 20, 25, and 30 figs of each breeding type, and then conducted chi-square and Fisher exact tests on each pseudosample to determine if there were differences in the occurrence of multiple pollinator species between monoecious and dioecious figs. Less than 3% of the pseudosamples showed significant differences in the occurrence of multiple pollinator species between fig breeding systems. The data were also analyzed by geographic region because almost all dioecious figs are only found in the Asian-Australasian region (Berg 1989) and none of the few dioecious species found in Africa have been surveyed. The incidence of copollinators in both breeding systems is still similar if one only considers Asia–Australasia (χ^2^ = 1.56, *P* = 0.2110; Fisher's *P* = 0.2895) or America–Asia–Australasia (χ^2^ = 0.18, *P* = 0.6685; Fisher's *P* = 0.8184). The incidence is barely different for samples from Africa–Asia–Australasia (χ^2^ = 3.86, *P* = 0.0493; Fisher's *P* = 0.0572) due to a smaller fraction of copollinators observed in monoecious (15/64 species) than dioecious figs (13/30 species), contrary to previous suggestions.

We find that pollinator host switches are significantly more common in monoecious than dioecious figs. In fact, the available data for the 119 species surveyed suggest that successful pollinator host switches have only occurred in monoecious figs (Table1 and Table S3). The proportion of copollinators that are sister species is significantly higher in dioecious figs (100%) than in monoecious figs (32.1%; χ^2^ = 16.44, *P* < 0.0001; Fisher's *P* < 0.0001). The latter result does not change when the copollinator phylogenetic data are analyzed using the minimum estimate of within-host duplications and host switches: no host switches are observed in dioecious figs, and the proportion of duplications is much higher in dioecious figs (100%) than in monoecious figs (43.9%; χ^2^ = 16.55, *P* < 0.0001; Fisher's *P* < 0.0001). Analyzing the data by geographic region, we see that the results are highly significant for America–Asia–Australasia (χ^2^ = 12.22, *P* = 0.0002; Fisher's *P* = 0.0003) and Africa–Asia–Australasia (χ^2^ = 9.71, *P* = 0.0009; Fisher's *P* = 0.0021), and still significant although not as highly so if one only considers Asia–Australasia (χ^2^ = 3.178, *P* = 0.0373; Fisher's *P* = 0.1558). The same results are obtained when we analyze the minimum estimate of within-host duplications and host switches by geographic region: America–Asia–Australasia (χ^2^ = 13.55, *P* < 0.0001; Fisher's *P* < 0.0001), Africa–Asia–Australasia (χ^2^ = 10.15, *P* = 0.0007; Fisher's *P* = 0.001), Asia–Australasia (χ^2^ = 2.96, *P* = 0.0427; Fisher's *P* = 0.1677).

**Table 1:** Summary of copollinator data from the literature (see Table S3)

	Monoecious			Dioecious		
	Sister species	Nonsister species	Total	Sister species	Nonsister species	Total
Multiple	9	19	28	13	0	13
Single	–	–	61	–	–	17
	Monoecious			Dioecious		
	Duplications	Host switching	Total	Duplications	Host switching	Total
Multiple	18	23	41	18	0	18
Single	–	–	61	–	–	17

The top section summarizes the phylogenetic relationships of copollinators (sister or nonsister species) for each fig breeding system. The bottom section shows the minimum number of duplication or host-switch events that can explain phylogenetic relationships among copollinators (see Materials and Methods for details).

It is important to note that the overall strong patterns we describe still stand even if we categorize *F. auriculata* and *F. oligodon* as a single species (incidence of copollinators in dioecious vs. monoecious figs: χ^2^ = 0.96, *P* = 0.3271; Fisher's *P* = 0.3698; proportion of copollinators that are sister species in dioecious vs. monoecious figs: χ^2^ = 15.51, *P* < 0.0001; Fisher's *P* < 0.0001), and if we remove the Mexican study of *F. microcarpa* (Su et al. 2008) given that this is an introduced species that has acquired two new pollinator species from a different genus in its new geographic range (Ramirez 1988; copollinator incidence in dioecious vs. monoecious figs: χ^2^ = 1.12, *P* = 0.2892; Fisher's *P* = 0.3642; proportion of copollinators that are sister species in dioecious vs. monoecious figs: χ^2^ = 14.86, *P* < 0.0001; Fisher's *P* < 0.0001).

## Discussion

### Widespread Violation of the One-Fig-One-Pollinator Rule

Figs and their pollinating wasps constitute perhaps the most tightly integrated pollination mutualism known. Molecular studies from the last 10 years have shown that a significant fractions of fig species (approximately 30%) are associated with more than one species of sympatric pollinator (Table1 and Table S3). In this study, we present additional evidence challenging the classic one-fig-to-one-pollinator paradigm. First, we present new empirical data from 15 fig species from Southwest China, showing the presence of copollinators in five of those species (Fig. 1). Further, we present meta-analyses of published molecular data from 119 fig species, concluding that 34.5% (41/119) of those fig species have multiple pollinator species. We stress the fact that although the fraction of the approximately 750 described *Ficus* species that have been properly surveyed for copollinators is close to 20%, the proportion of fig species with multiple pollinator species is likely to be an underestimate because the more intensely that a fig tree species is studied, the more likely that additional pollinator species will be detected (Compton and Hawkins 1992).

This study presents the first formal test of the suggestion that the occurrence of copollinators may be influenced by the fig breeding system (Cook and Segar 2010; Moe et al. 2011). Contrary to previous suggestions, our meta-analyses of molecular studies show that the incidence of copollinators in dioecious and monoecious figs is similar: copollinators have been found in 31.5% of 89 monoecious fig species, and in 43.3% of 30 dioecious fig species. Further, we show that biased geographic sampling cannot explain the results because patterns are the same even if different geographic regions are analyzed separately. Additional broad scale surveys are needed to more precisely quantify the incidence of the breakdown of the one-fig-to-one-pollinator rule.

### Pollinator Host-Specificity and Speciation in Dioecious and Monoecious Figs

The most remarkable result we report from the meta-analysis is the observation of a significant difference in the phylogenetic relationships of copollinators between the two fig breeding systems. In monoecious figs, copollinators are either sister species (9/28 species with multiple pollinator species), reflecting speciation within the same host fig (duplication), or, in most cases, they are unrelated species (19/28), reflecting host switches (Table1). In dioecious figs, on the other hand, copollinators are only sister species (13/13 species; Table1). This result is not due to biased geographic sampling, because the result is the same even if different geographic regions are analyzed separately. This remarkable result suggests that there are major differences in the mechanisms controlling patterns of fig wasp host fidelity depending on the fig breeding system: species isolation mechanisms seem to be stronger in dioecious figs and/or pollinator host specificity is higher in dioecious figs. Moreover, these results further support the conclusion that strict cospeciation between figs and their pollinators may still be a valid paradigm for dioecious (Weiblen and Bush 2002; Moe et al. 2011) but not for monoecious figs (Machado et al. 2005; Herre et al. 2008; Jackson et al. 2008), and if that is the case we may need to modify our current models of fig–fig wasp evolution to include the evolutionary consequences of diffuse coevolution on patterns of fig genetic diversity (Machado et al. 2005; Herre et al. 2008; Cook and Segar 2010).

What could explain the observation that pollinator host switches are common in monoecious figs, but are not observed in dioecious figs? The differences in dispersal ecology between pollinators from dioecious and monoecious *Ficus* could be an important part of the explanation (Compton et al. 2000; Harrison 2003; Harrison and Rasplus 2006). However, this cannot be the sole reason because we know there is strong host fidelity in a large fraction of species that should be under the control of robust species-recognition mechanisms that greatly reduce host identification mistakes and host switches. We therefore propose two nonmutually exclusive hypotheses: (1) There is stronger premating isolation (i.e., higher pollinator specificity) in dioecious than monoecious figs. Therefore, the probability of host identification mistakes is much lower in pollinators of dioecious figs. (2) Fitness costs experienced by pollinators that make host identification mistakes are greater in dioecious than monoecious figs. Therefore, there is a lower probability of successful host switches in pollinators of dioecious figs.

Under the first hypothesis, we propose that strong fig premating isolation can be the result of strong disruptive selection between dioecious fig species in the composition of volatiles released for long-range attraction of pollinators or in the composition of chemical cues in the syconium or ostiole surface for short-range attraction of pollinators. Species specificity in the fig–fig wasp mutualism is the result of species-specific volatile signals released by figs (Van Noort et al. 1989; Chen et al. 2009; Hossaert-McKey et al. 2010; Wang et al. 2013), although tactile chemical cues also seem to be important (Wang et al. 2013). Strong differentiation of volatiles minimizes the likelihood of host identification mistakes by fig wasps and can therefore constitute a very effective fig reproductive barrier. A simple testable prediction of our first hypothesis is that there should be more pronounced differences in the volatile profiles of closely related and sympatric dioecious than monoecious fig species, reflecting strong disruptive selection in pollinator attractants. Although there are no studies addressing this specific hypothesis, comparative studies of fig volatile profiles from about 40 different fig species have been published (Hossaert-McKey et al. 2010). In dioecious figs there is evidence of strong differences in volatile profiles between closely related species (Proffit et al. 2009) or varieties of the same species (Chen et al. 2009; Wang et al. 2013). Those studies and others also show strong evidence of chemical mimicry between receptive male and female figs (Hossaert-McKey et al. 2010). Studies in monoecious figs have shown that some fig pollinating wasps can be attracted to figs species other than their typical hosts (Grison-Pige et al. 2002), and a more recent study (Cornille et al. 2012) has shown that the volatile profiles of *Ficus natalensis* and *F. burkei*, two African monoecious fig species that share one species of pollinator (*Elisabethiella stuckenbergi*), are very similar but differ from the volatile profiles of other African species. The similarity in volatiles may explain pollinator sharing between the two fig species (Cornille et al. 2012). Although the available evidence seems to support our hypothesis, more comparative studies are needed to directly test it.

Under the second hypothesis, pollinators entering a syconium from the wrong fig host pay much higher fitness costs in dioecious than in monoecious figs. There would thus be strong selection to minimize host identification mistakes in the pollinators of dioecious figs, which generates a much lower rate of novel host colonization events. Compared with the pollinators of monoecious figs, pollinators of dioecious figs may be under strong selection to find figs that are similar to their birth figs given the large fitness cost paid by pollen-bearing wasps entering a female syconium even if it is from the correct host species (Janzen 1979). In monoecious figs, on the other hand, those fitness costs may be lower, facilitating the occurrence of host switches. A similar idea has been previously described in the context of explaining suggested differences in copollinator frequency between the two breeding systems (Moe et al. 2011). Our hypothesis, however, further predicts that a higher proportion of sister copollinator species should be observed in dioecious figs, given that speciation within the same fig host is thus more likely than speciation caused by switching hosts. Although the actual mechanism(s) by which pollinator speciation within the same host fig could occur are still unknown, a pattern of copollinators being mostly sister species could be generated by their highly structured and inbred population structures that can reinforce rapid genetic divergence leading to speciation (Machado et al. 2005), and/or by allopatric divergence or pollinator populations followed by secondary contact (Cook and Segar 2010; Chen et al. 2011; Wei et al. 2014). The later scenario seems more likely in dioecious figs given the shorter dispersal ranges in pollinators of this breeding system (Compton et al. 2000; Harrison 2003; Harrison and Rasplus 2006; Nazareno and Carvalho 2009).

A simple testable prediction of the second hypothesis is that one should observe significant fitness reduction in pollinators colonizing the wrong host in dioecious species, but less so in monoecious species. That pattern should be the result of the evolution of mechanisms that inhibit the development of pollinators arriving with heterospecific pollen. Results from three recent experimental manipulation studies in dioecious figs are consistent with the prediction. One large study (Moe and Weiblen 2012) compared the effects of heterospecific and conspecific pollination of male and female figs of *Ficus hispidioides* by pollinators from four different close relatives. The results of those experiments clearly show that although heterospecific pollinators introduced in male figs of *F. hispidioides* could oviposit, they could not develop, underscoring the strong fitness cost paid by pollinators trying to complete their life cycles in the wrong dioecious fig host. Those results suggest that there should be strong selection for recognizing the correct fig host (Moe et al. 2011). Interestingly, female figs pollinated by heterospecific pollinators produced viable hybrid seedlings although not in every species (Moe and Weiblen 2012), suggesting that fig interspecific hybridization is possible even in the absence of successful pollinator colonization. Another study using experimental manipulations in *F. semicordata* (Wang et al. 2013) showed that abortion rates of seed figs are higher and the viability of seedlings is lower when pollination is performed by wasps carrying pollen from a different *F. semicordata* variety, suggesting stronger postzygotic isolation mechanisms in this species than in *F. hispidioides*. The third study conducted reciprocal manipulation experiments with the pollinators of two closely related species, *F. auriculata* and *Ficus hainanensis* (Yang et al. 2012), finding that abortion rates of figs pollinated with heterospecific pollen are asymmetric (higher in *F. auriculata*). Interestingly, the two pollinators can still develop in the wrong fig host although a clear trade-off between progeny number and size was observed. Finally, although there are no published studies in monoecious figs that rely on experimental manipulations, data comparing the performance of a pollinator shared by two Neotropical fig hosts show no detectable differences in lifetime reproductive success or seed production (E. A. Herre, pers. comm.). There is thus a clear need to increase the number of studies using experimental manipulations.

### Consequences for Strict Cospeciation

The evidence we present suggests that we need to modify our current models of fig–fig wasp coevolution to include the evolutionary consequences of less strict cospeciation on patterns of fig genetic diversity, particularly in monoecious figs. Several reported cases of interspecific or intermorphotype hybridization in dioecious figs have been described (Ramirez 1994; Parrish et al. 2003; Wei et al. 2014). However, genetic and pollinator manipulation studies have not found any significant evidence of introgression even if hybridization was possible in a study of six sympatric dioecious species (Moe and Weiblen 2012) or in a study of three interfertile morphotypes of the *F. auriculata* species complex that share pollinators (Wei et al. 2014). On the other hand, genetic evidence, albeit more limited, has shown signals of introgression between sympatric monoecious fig species (Machado et al. 2005; Jackson et al. 2008; Renoult et al. 2009). Overall, our current knowledge regarding evidence of introgression and phylogenetic relationships among copollinators is consistent with differences in the observed level of congruency between the phylogenies of figs and their pollinators depending on the fig breeding system. Congruent phylogenetic patterns have been observed between dioecious figs and their pollinators (Weiblen and Bush 2002; Weiblen 2004; Cruaud et al. 2012), suggesting that strict cospeciation is more common in that interaction (Moe et al. 2011). That conclusion is fully supported by predictions generated by our meta-analyses. On the other hand, incongruent phylogenetic patterns between monoecious figs and their pollinators (Machado et al. 2005; Cruaud et al. 2012) are consistent with conclusions from our meta-analyses, showing that host switches are more common in that breeding system. Future studies of cospeciation or interspecific hybridization should take into account this clear difference between the two breeding systems.

## Data Archiving

Sequences were deposited in GenBank (accession numbers HM802558-HM802653, HM802669-HM802758, HQ456879). The doi for our data is 10.5061/dryad.7613f.

## Supporting Information

**Figure S1**. The *COI* phylogenetic tree of the surveyed fig pollinating wasps. **Figure S2**. The 28S rRNA phylogenetic tree of the surveyed fig pollinating wasps. **Figure S3**. The *COI* Bayesian phylogeny of the genus *Ceratosolen*. **Figure S4**. The *COI* Bayesian phylogeny of the genus *Eupristina*. **Figure S5**. The *COI* Bayesian phylogeny of *Ceratosolen gravelyi* and *C. emarginatus*. **Table S1**. The fig wasp and fig species investigated. **Table S2**. Literature search result of 22 key word combinations. **Table S3**. Fig wasps and host fig trees involved in the meta-analyses. **Table S4**. Summary of copollinator data from the literature, analyzed by geographic region.
