# Supplementary material for: The incidence and pattern of copollinator diversification in dioecious and monoecious figs
Source: Evolution. 2015 Jan 19;69(2):294–304. doi: 10.1111/evo.12584 (PMC4328460; doi:10.1111/evo.12584)
Supplement: Supplementary file 1 — Figure S1. The COI phylogenetic tree of the surveyed fig pollinating wasps. Figure S2. The 28S rRNA phylogenetic tree of the surveyed fig pollinating wasps. Figure S3. The COI Bayesian phylogeny of the genus Ceratosolen. Figure S4. The COI Bayesian phylogeny of the genus Eupristina. Figure S5. The COI Bayesian phylogeny of Ceratosolen gravelyi and C. emarginatus. Table S1. The fig wasp and fig species investigated. Table S2. Literature search result of 22 key word combinations. Table S3. Fig wasps and host fig trees involved in the meta-analyses. Table S4. Summary of copollinator data from the literature, analyzed by geographic region. [file evo0069-0294-sd1.zip › evo12584-sup-0001-SupInfo/evo12584-sup-0001-figureS1-S5.pdf]

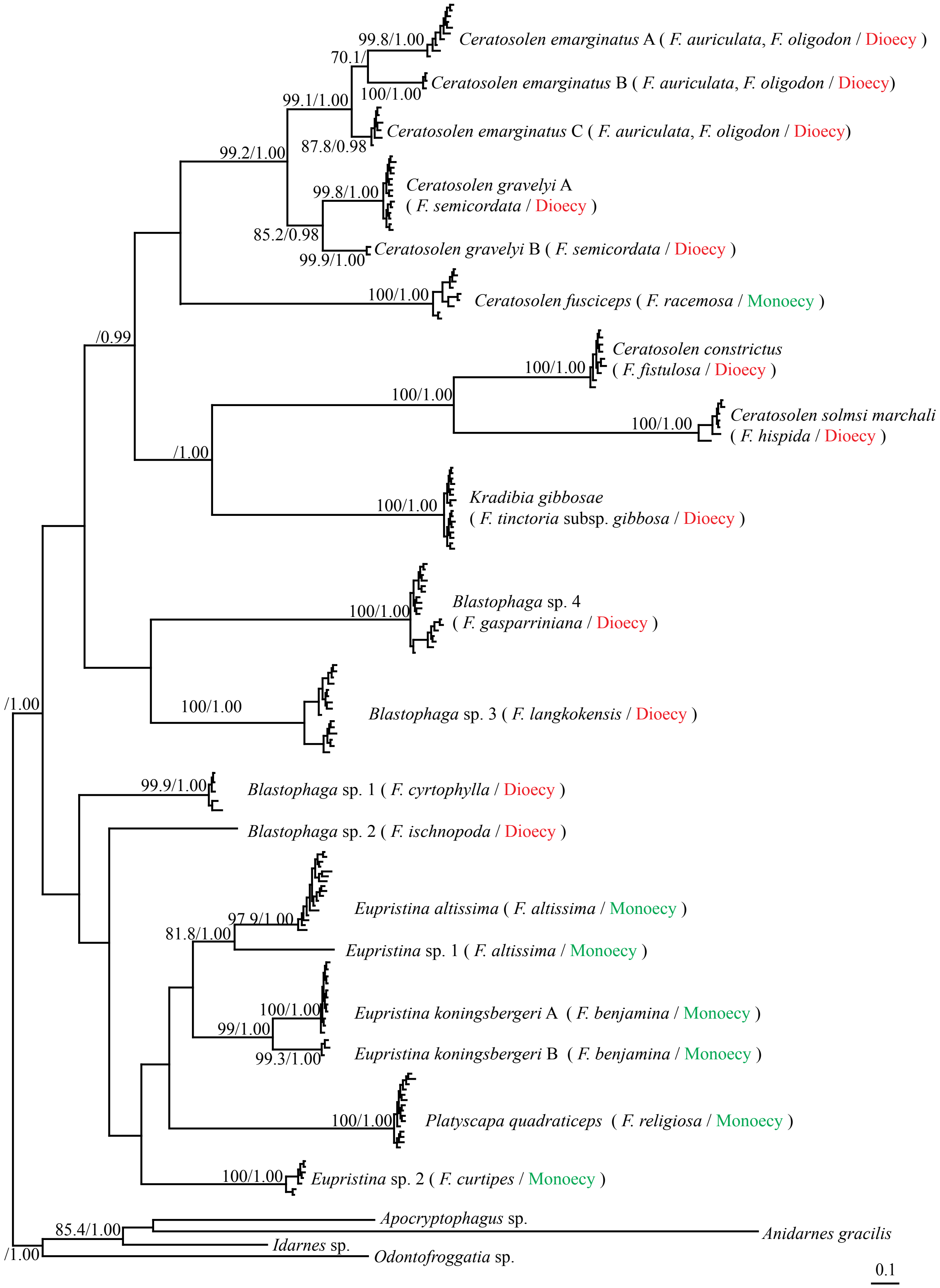
99.8/1.00
70.1/
Ceratosolen emarginatus A (F. auriculata, F. oligodon / Dioecy)
100/1.00
Ceratosolen emarginatus B (F. auriculata, F. oligodon / Dioecy)
99.1/1.00
87.8/0.98
Ceratosolen emarginatus C (F. auriculata, F. oligodon / Dioecy)
99.2/1.00
99.8/1.00
Ceratosolen gravelyi A
(F. semicordata / Dioecy)
85.2/0.98
99.9/1.00
Ceratosolen gravelyi B (F. semicordata / Dioecy)
100/1.00
Ceratosolen fusciceps (F. racemosa / Monoecy)
/0.99
100/1.00
Ceratosolen constrictus
(F. fistulosa / Dioecy)
100/1.00
100/1.00
Ceratosolen solmsi marchali
(F. hispida / Dioecy)
/1.00
100/1.00
Kradibia gibbosae
(F. tinctoria subsp. gibbosa / Dioecy)
100/1.00
Blastophaga sp. 4
(F. gasparriniana / Dioecy)
/1.00
100/1.00
Blastophaga sp. 3 (F. langkokensis / Dioecy)
99.9/1.00
Blastophaga sp. 1 (F. cyrtophylla / Dioecy)
Blastophaga sp. 2 (F. ischnopoda / Dioecy)
97.9/1.00
Eupristina altissima (F. altissima / Monoecy)
81.8/1.00
Eupristina sp. 1 (F. altissima / Monoecy)
100/1.00
Eupristina koningsbergeri A (F. benjamina / Monoecy)
99/1.00
99.3/1.00
Eupristina koningsbergeri B (F. benjamina / Monoecy)
100/1.00
Platyscapa quadraticeps (F. religiosa / Monoecy)
100/1.00
Eupristina sp. 2 (F. curtipes / Monoecy)
85.4/1.00
Apocryptophagus sp.
Anidarnes gracilis
Idarnes sp.
/1.00
Odontofroggatia sp.
0.1

Figure S1. The COI Bayesian tree of fig pollinating wasps collected from 15 host figs. ML bootstrap percentages (>70%. 1000 replications) and Bayesian posterior probabilities (> 0.95, 107 generations) are indicated at the nodes. Fig host names are in parentheses and breeding system of the fig host is also indicated.

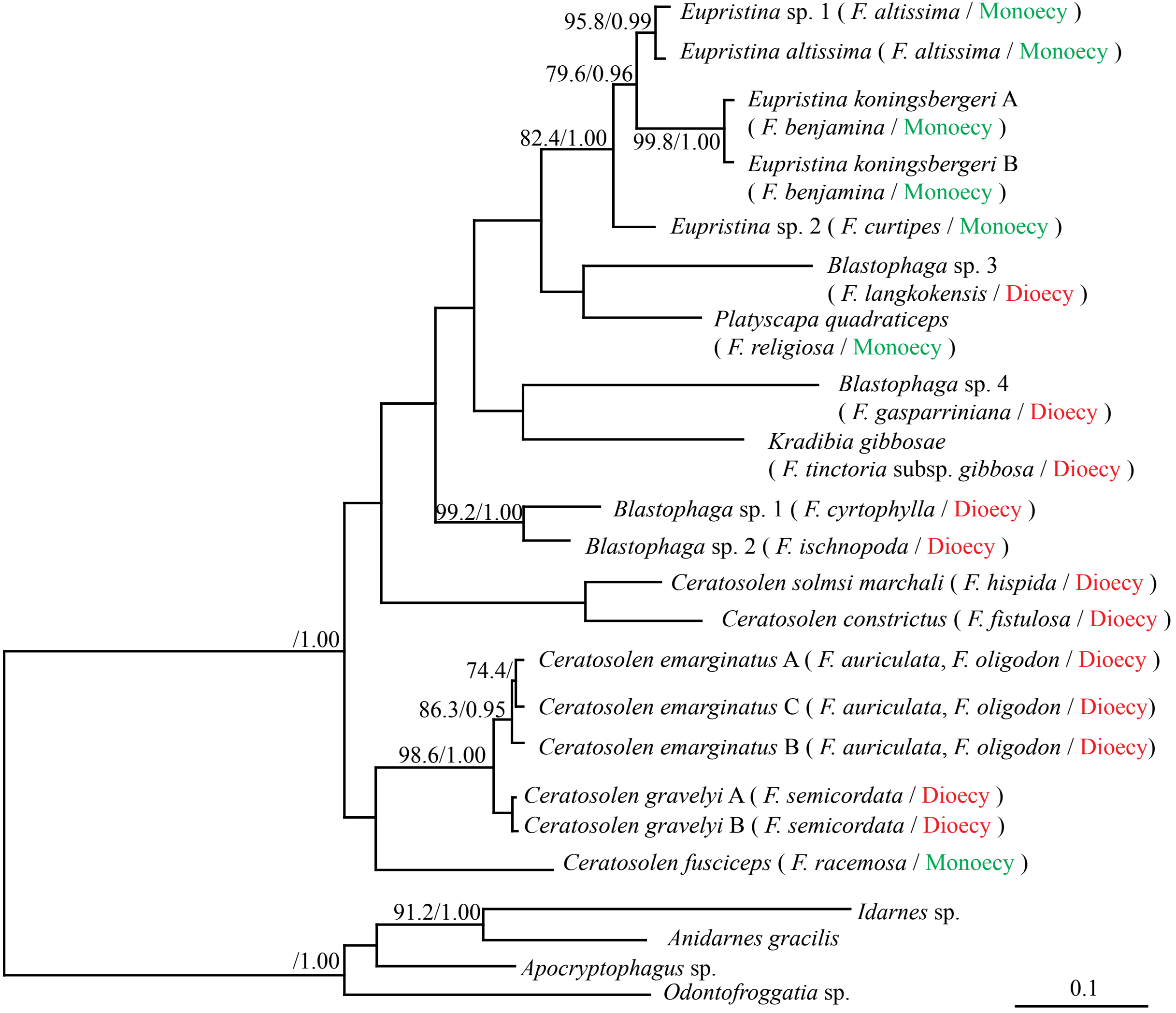
95.8/0.99
Eupristina sp. 1 (F. altissima / Monoecy)
Eupristina altissima (F. altissima / Monoecy)
79.6/0.96
Eupristina koningsbergeri A (F. benjamina / Monoecy)
82.4/1.00
99.8/1.00
Eupristina koningsbergeri B (F. benjamina / Monoecy)
Eupristina sp. 2 (F. curtipes / Monoecy)
Blastophaga sp. 3 (F. langkokensis / Dioecy)
Platyscapa quadraticeps (F. religiosa / Monoecy)
Blastophaga sp. 4 (F. gasparriniana / Dioecy)
Kradibia gibbosae (F. tinctoria subsp. gibbosa / Dioecy)
99.2/1.00
Blastophaga sp. 1 (F. cyrtophylla / Dioecy)
Blastophaga sp. 2 (F. ischnopoda / Dioecy)
Ceratosolen solmsi marchali (F. hispida / Dioecy)
/1.00
Ceratosolen constrictus (F. fistulosa / Dioecy)
74.4/
Ceratosolen emarginatus A (F. auriculata, F. oligodon / Dioecy)
86.3/0.95
Ceratosolen emarginatus C (F. auriculata, F. oligodon / Dioecy)
Ceratosolen emarginatus B (F. auriculata, F. oligodon / Dioecy)
98.6/1.00
Ceratosolen gravelyi A (F. semicordata / Dioecy)
Ceratosolen gravelyi B (F. semicordata / Dioecy)
Ceratosolen fusciceps (F. racemosa / Monoecy)
91.2/1.00
Idarnes sp.
Anidarnes gracilis
/1.00
Apocryptophagus sp.
Odontofroggatia sp.
0.1

Figure S2. The 28S rRNA Bayesian tree of fig pollinating wasps collected from 15 host figs. ML bootstrap percentages (>70%. 1000 replications) and Bayesian posterior probabilities (> 0.95, 107 generations) are indicated at the nodes. Fig host names are in parentheses and breeding system of the fig host is also indicated.

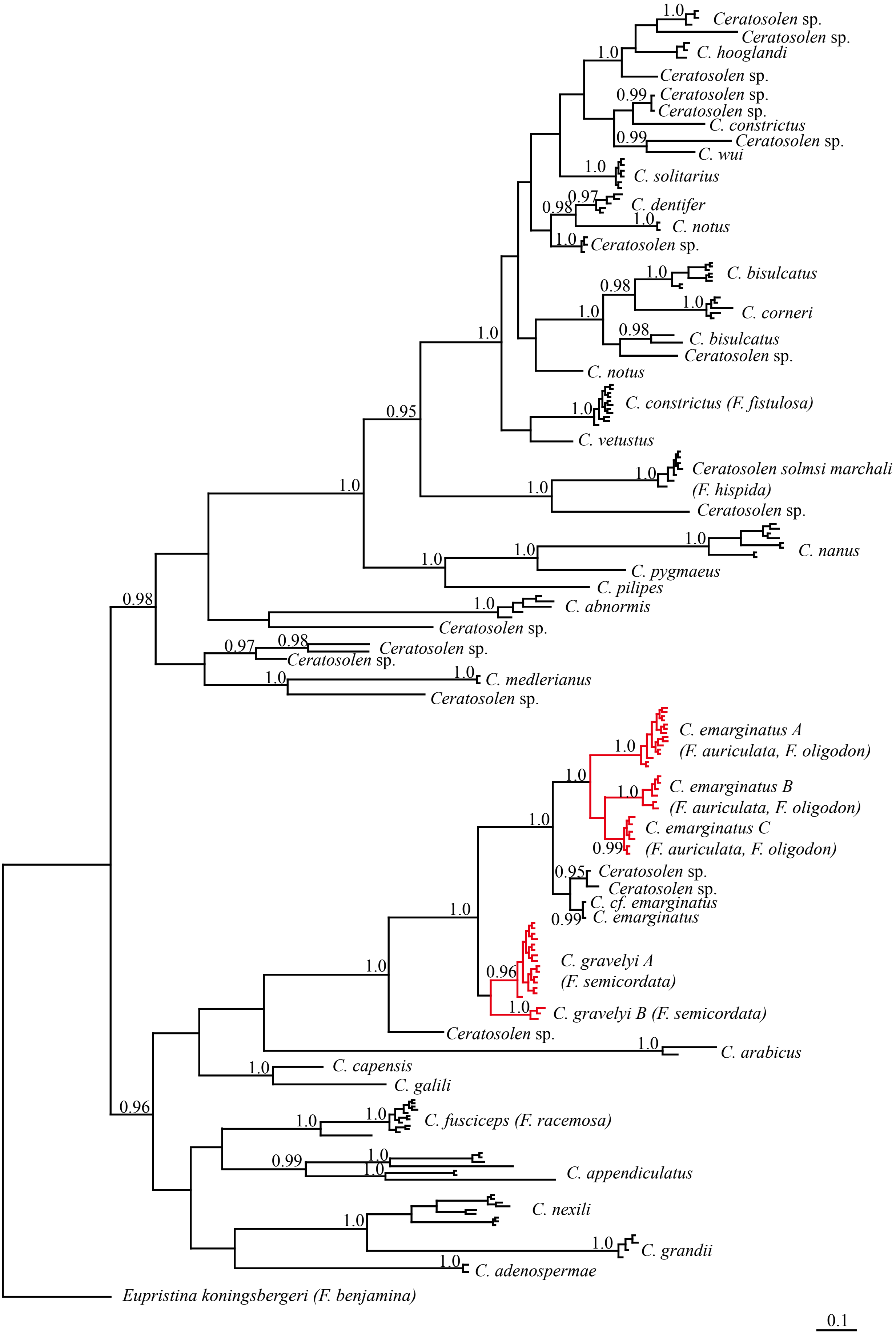

1.0
Ceratosolen sp.
Ceratosolen sp.
C. hooglandi
1.0
Ceratosolen sp.
0.99
Ceratosolen sp.
Ceratosolen sp.
C. constrictus
0.99
Ceratosolen sp.
C. wui
1.0
C. solitarius
0.97
0.98
C. dentifer
1.0
C. notus
1.0
Ceratosolen sp.
1.0
C. bisulcatus
0.98
1.0
C. corneri
1.0
0.98
C. bisulcatus
Ceratosolen sp.
1.0
C. notus
1.0
C. constrictus (F. fistulosa)
C. vetustus
0.95
1.0
Ceratosolen solmsi marchali
(F. hispida)
1.0
1.0
Ceratosolen sp.
1.0
C. nanus
1.0
1.0
C. pygmaeus
C. pilipes
0.98
1.0
C. abnormis
Ceratosolen sp.
0.97
0.98
Ceratosolen sp.
Ceratosolen sp.
1.0
1.0
C. medlerianus
Ceratosolen sp.
1.0
C. emarginatus A
(F. auriculata, F. oligodon)
1.0
1.0
C. emarginatus B
(F. auriculata, F. oligodon)
1.0
C. emarginatus C
0.99
(F. auriculata, F. oligodon)
0.95
Ceratosolen sp.
Ceratosolen sp.
C. cf. emarginatus
0.99
C. emarginatus
1.0
C. gravelyi A
1.0
0.96
(F. semicordata)
1.0
C. gravelyi B (F. semicordata)
Ceratosolen sp.
1.0
C. arabicus
1.0
C. capensis
C. galili
0.96
1.0
1.0
C. fusciceps (F. racemosa)
0.99
1.0
1.0
C. appendiculatus
C. nexili
1.0
1.0
C. grandii
1.0
C. adenospermae
Eupristina koningsbergeri (F. benjamina)
0.1

Figure S3. The COI Bayesian tree of the genus *Ceratosolen* with the posterior probabilities (> 0.95, 107 generations). The tree includes sequences from all the additional *Ceratosolen* species available in GenBank. The multiple pollinators of *C. emarginatus* and *C. gravelyi* from the present study are highlighted in red.

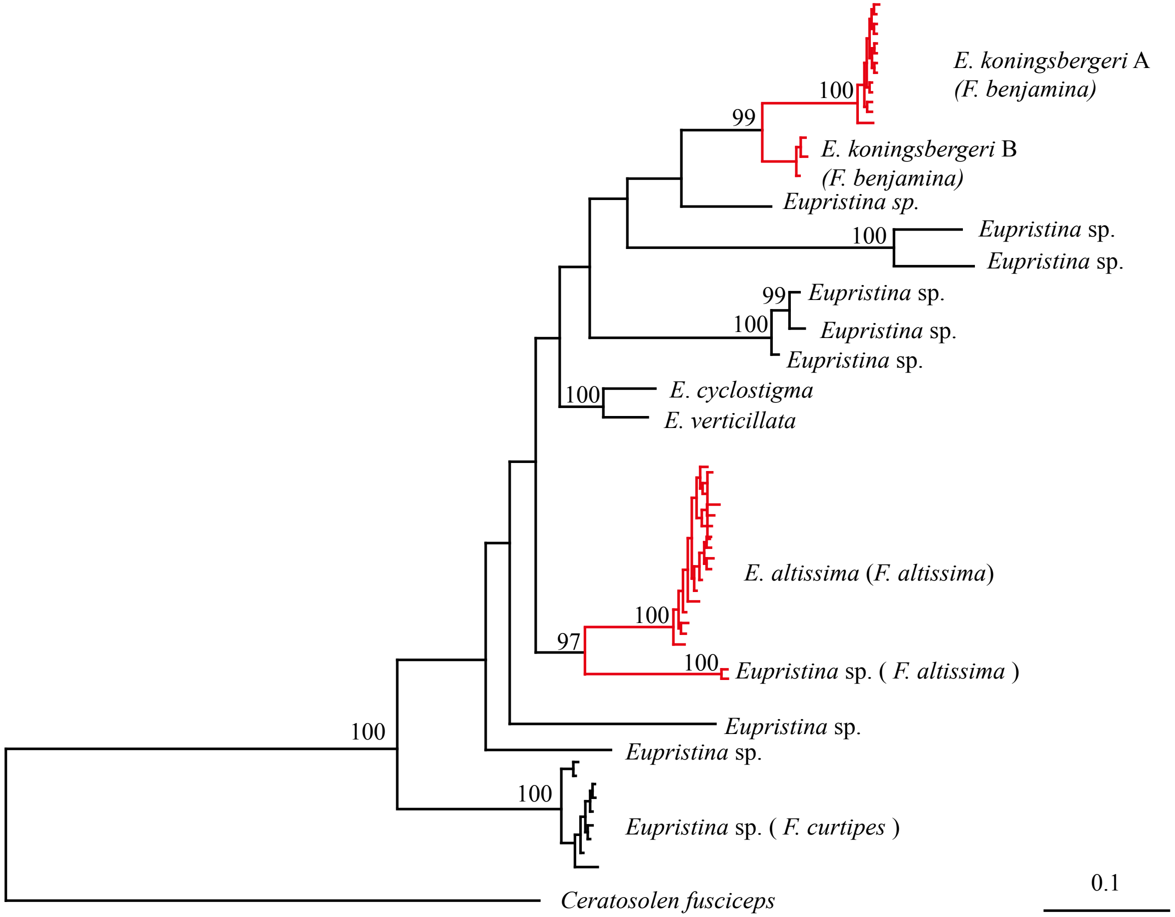

E. koningsbergeri A
(F. benjamina)
100
99
E. koningsbergeri B
(F. benjamina)
Eupristina sp.
100
Eupristina sp.
Eupristina sp.
99
Eupristina sp.
100
Eupristina sp.
Eupristina sp.
100
E. cyclostigma
E. verticillata
E. altissima (F. altissima)
100
97
100
Eupristina sp. (F. altissima)
Eupristina sp.
100
Eupristina sp.
100
Eupristina sp. (F. curtipes)
Ceratosolen fusciceps
0.1

Figure S4. The COI Bayesian tree of the genus *Eupristina* with the posterior probabilities (> 0.95, 107 generations). The tree includes sequences from all the additional *Eupristina* species available in GenBank. The multiple pollinators of *E. altissima* and *E. koningsbergeri* from the present study are highlighted in red.

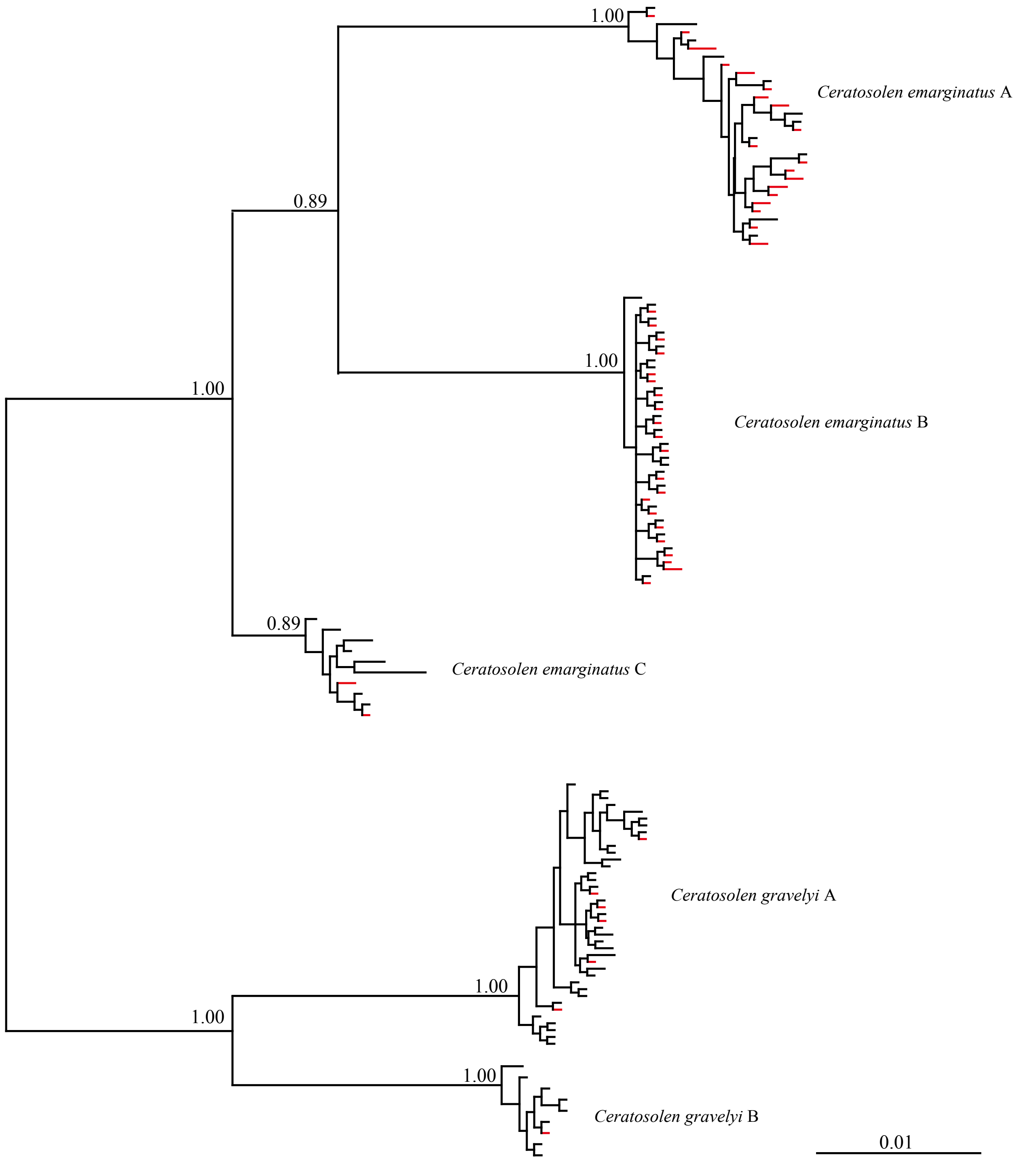
1.00
0.89
1.00
1.00
0.89
1.00
1.00
1.00
Ceratosolen emarginatus A
Ceratosolen emarginatus B
Ceratosolen emarginatus C
Ceratosolen gravelyi A
Ceratosolen gravelyi B
0.01

Figure S5. The COI Bayesian tree of *Ceratosolen gravelyi* and *Ceratosolen emarginatus* with the posterior probabilities (> 0.95, 10 generations). Wasp individuals that were collected emerging from syconia are highlighted in red, while foundress wasps are highlighted in black.
