# Supplementary material for: The incidence and pattern of copollinator diversification in dioecious and monoecious figs
Source: Evolution. 2015 Jan 19;69(2):294–304. doi: 10.1111/evo.12584 (PMC4328460; doi:10.1111/evo.12584)
Supplement: Supplementary file 1 — Figure S1. The COI phylogenetic tree of the surveyed fig pollinating wasps. Figure S2. The 28S rRNA phylogenetic tree of the surveyed fig pollinating wasps. Figure S3. The COI Bayesian phylogeny of the genus Ceratosolen. Figure S4. The COI Bayesian phylogeny of the genus Eupristina. Figure S5. The COI Bayesian phylogeny of Ceratosolen gravelyi and C. emarginatus. Table S1. The fig wasp and fig species investigated. Table S2. Literature search result of 22 key word combinations. Table S3. Fig wasps and host fig trees involved in the meta-analyses. Table S4. Summary of copollinator data from the literature, analyzed by geographic region. [file evo0069-0294-sd1.zip › evo12584-sup-0001-SupInfo/evo12584-sup-0005-tableS4.docx]

**Table S4**. Summary of co-pollinator data from the literature, analyzed by geographic region

|  | Africa (Monoecious) | America (Monoecious) | Asia (Dioecious) | Asia (Monoecious) | Summary |
| --- | --- | --- | --- | --- | --- |
| Fig numbers | 32 | 25 | 30 | 32 | 119 |
| Multiple | 6 | 13 | 13 | 9 | 41 |
| Single | 26 | 12 | 17 | 23 | 78 |
| Sister | 0 | 2 | 13 | 7 | 22 |
| Non-sister | 6 | 11 | 0 | 2 | 19 |
| Host-switches | 9 | 12 | 0 | 2 | 23 |
| Duplication | 4 | 3 | 18 | 11 | 36 |
